# What works in radiology education for medical students: a systematic review and meta-analysis

**DOI:** 10.1186/s12909-023-04981-z

**Published:** 2024-01-10

**Authors:** Stuart W.T. Wade, Gary M. Velan, Nicodemus Tedla, Nancy Briggs, Michelle Moscova

**Affiliations:** 1 Westmead Hospital, Sydney, Australia; 2 School of Biomedical Sciences, Faculty of Medicine & Health, The University of New South Wales, Sydney, Australia; 3 Office of Medical Education, The University of New South Wales, Sydney, Australia; 4 Stats Central, Mark Wainwright Analytical Centre, The University of New South Wales, Sydney, Australia

**Keywords:** Undergraduate radiology education, Medical student radiology education, Medical student education, Medical imaging education, Radiology teaching

## Abstract

**Background:**

Medical imaging related knowledge and skills are widely used in clinical practice. However, radiology teaching methods and resultant knowledge among medical students and junior doctors is variable. A systematic review and meta-analysis was performed to compare the impact of different components of radiology teaching methods (active versus passive teaching, eLearning versus traditional face-to-face teaching) on radiology knowledge / skills of medical students.

**Methods:**

PubMed and Scopus databases were searched for articles published in English over a 15-year period ending in June 2021 quantitatively comparing the effectiveness of undergraduate medical radiology education programs regarding acquisition of knowledge and/or skills. Study quality was appraised by the Medical Education Research Study Quality Instrument (MERSQI) scoring and analyses performed to assess for risk of bias. A random effects meta-analysis was performed to pool weighted effect sizes across studies and I^2^ statistics quantified heterogeneity. A meta-regression analysis was performed to assess for sources of heterogeneity.

**Results:**

From 3,052 articles, 40 articles involving 6,242 medical students met inclusion criteria. Median MERSQI score of the included articles was 13 out of 18 possible with moderate degree of heterogeneity (I^2^ = 93.42%). Thematic analysis suggests trends toward synergisms between radiology and anatomy teaching, active learning producing superior knowledge gains compared with passive learning and eLearning producing equivalent learning gains to face-to-face teaching. No significant differences were detected in the effectiveness of methods of radiology education. However, when considered with the thematic analysis, eLearning is at least equivalent to traditional face-to-face teaching and could be synergistic.

**Conclusions:**

Studies of educational interventions are inherently heterogeneous and contextual, typically tailored to specific groups of students. Thus, we could not draw definitive conclusion about effectiveness of the various radiology education interventions based on the currently available data. Better standardisation in the design and implementation of radiology educational interventions and design of radiology education research are needed to understand aspects of educational design and delivery that are optimal for learning.

**Trial registration:**

Prospero registration number CRD42022298607.

**Supplementary Information:**

The online version contains supplementary material available at 10.1186/s12909-023-04981-z.

## Background

Diagnostic imaging interpretation is an essential skill for medical graduates, as imaging is frequently utilised in medical practice. However, radiology is often under-represented in medical curricula [1, 2]. Exposure to radiology education in medical school could result in better understanding of the role of imaging, leading to benefits such as enhanced selection of imaging, timely diagnosis and, subsequently, improved patient care [1]. There is no consensus as to how radiology should be taught in undergraduate medical programs, and methods vary widely across the globe [3–6]. Great diversity exists in radiology topics taught, the stage of learning at which radiology is introduced to students, and the training of those teaching radiology [7, 8]. In addition to traditional lectures, small group tutorials, case conferences or clerkship models, many newer methods of delivering radiology education have been described [1, 5, 9, 10]. These include eLearning, flipped classrooms and in diagnostic reasoning simulator programs [1, 11].

Radiology is particularly suited to eLearning, given the digitisation of medical imaging and its ease of incorporation into eLearning resources [1]. eLearning can provide easy access to radiology education, regardless of students’ location and has been increasingly utilized, particularly following the onset of the COVID-19 pandemic [10, 12–14]. In addition to delivery methods, other factors may influence effectiveness of radiology education, including active or passive method of instruction, instructor expertise and content complexity.

Many studies looked at individual educational interventions, typically confined to a single cohort with limited sample size, making it difficult to make recommendations on how radiology education should be delivered. Thus, we conducted a systematic review and meta-analysis to determine the factors associated with effective radiology knowledge or skill acquisition by undergraduate medical students. We analysed teaching methods, modes of delivery, instructor expertise, content taught, medical student experience / seniority, and the methods of assessment as outlined in Supplementary Material 1.

## Methods

This study utilised a prospectively designed protocol which is in concordance with the Preferred Reporting Items for Systematic Reviews and Meta-Analyses (PRISMA) Statement [15]. Ethics approval was not required as this is a systematic review and meta-analysis.

### Search identification

A systematic search strategy was designed to identify articles evaluating knowledge and / or skill acquisition following radiology education interventions for medical students. With the assistance of a university librarian, PubMed and Scopus databases were searched for articles dating from January 2006 to the time of review in June 2021 using the search terms listed in Table 1. Initial screening was performed by a single reviewer (SW), limiting the studies to articles written in English. Abstracts were screened and where ambiguity existed, the full articles were reviewed. Where full text articles were not available, the corresponding authors were contacted for a copy. Duplicate articles and those where full text versions could not be obtained were removed.

**Table 1:** Databases searched and search terms utilised

Database	Search terms
PubMed	(“medical students“[All Fields] OR undergraduate [All Fields]) AND (“radiology“[All Fields] OR “medical imaging“[All Fields]) AND (“education“[All Fields] OR “teaching“[All Fields] OR elearning[All Fields])
Scopus	(Medical student OR undergraduate) AND (Radiology OR medical imaging) AND (education OR teaching OR elearning) - (Limited to original articles only)

### Study eligibility and inclusion

The shortlisted articles were reviewed by two authors (SW, NT) according to the inclusion and exclusion criteria which are summarised in outlined in Table 2. The articles were discussed by the authors and where ambiguity existed, consensus was achieved following discussion with a third author (MM).Where missing data precluded calculation of the effect size of an educational intervention, several attempts were made to contact corresponding authors via email. If no response was received, the article was excluded. Cohen’s D effect sizes were calculated from available data, then independently reviewed by a statistician.

**Table 2:** Inclusion and exclusion criteria for undergraduate medical student radiology education intervention research

Criteria	Inclusion	Exclusion
Population	Radiology education involving undergraduate medical students.	Radiology education in non-medical professions or post graduate doctors only.
Intervention	Original research of existing educational programs where objective assessment of radiology knowledge has occurred. Medical imaging education programs addressing indications, risks, contraindications and interpretation of imaging in humans only. Medical imaging education as part of anatomy or surgical education where radiological components are taught and/ or examined.	Original research with subjective assessment only such as educators and or students’ perspectives. Original research without comparison to a control group. Original research focusing on nuclear medicine, interventional radiology, cardiac ultrasound or dental radiology teaching or physics of imaging. Programs where data required to calculate effect sizes was not available in the original publication and after attempts to contact the corresponding authors.
Comparison	Comparison between methods of delivery of radiology education and their effectiveness on radiology knowledge and or skill acquisition.	
Outcome	The primary outcome measure is a comparison of calculated effect sizes based on results in post intervention tests of radiology knowledge and or skills assessments.	

### Quality assessment

The methodological quality of all articles meeting the inclusion criteria were quantitatively assessed using the Medical Education Research Study Quality Instrument (MERSQI) [16]. Risk of bias was assessed according to the Cochrane collaboration risk of bias assessment tools: Revised Cochrane risk-of-bias for randomized trials (RoB 2) [17] and Risk of Bias in Non-randomized Studies of Interventions (ROBINS-1) [18]. Tabulated representations were constructed using the Risk-of-bias visualization (robvis) [19] package.

### Data extraction

Data extracted included publication details, sample sizes, medical students’ seniority, instructor expertise, educational delivery methods, radiology content and methods of assessment. A more comprehensive description can be found in Supplementary Material (1) Extracted data points are defined in Supplementary Material (2) Cohen’s D effect sizes were recorded when published or calculated from available data.

Many studies employed several methods of educational delivery. If the intervention group received an educational resource (e.g. eLearning) in addition to an educational activity shared by both intervention and control groups (e.g. lecture), then only the additional activity (i.e. eLearning) was included in the comparison analysis. When two reviewers were undecided about how to classify data extracted from a study, the outcome would be resolved by consensus after review by a third author (MM). If disagreement remained, attempts to contact the authors for additional information were made. If a final determination was unable to be made, the study was excluded.

### Data synthesis

A random effects meta-analysis model was used to obtain the pooled estimate of the standardised mean difference (SMD) based on Cohen’s D effect size calculations. Heterogeneity was quantified by I² statistics, which estimate the percentage of variability across studies not due to chance. Evidence of publication bias was assessed by visual inspection of funnel plots and regression tests. A meta-regression analysis was performed to examine the possible sources of heterogeneity and the association between study factors and the intervention effect (SMD). All statistical analyses were performed with R version 4.1.2 (R Foundation for Statistical Computing Vienna Austria) using the R package metafor 2010.

All effect sizes were expressed as Cohen’s D which were interpreted as 0.2 for small, 0.5 for moderate and ≥ 0.8 as a large effect size.

## Results

**Fig. 1:**
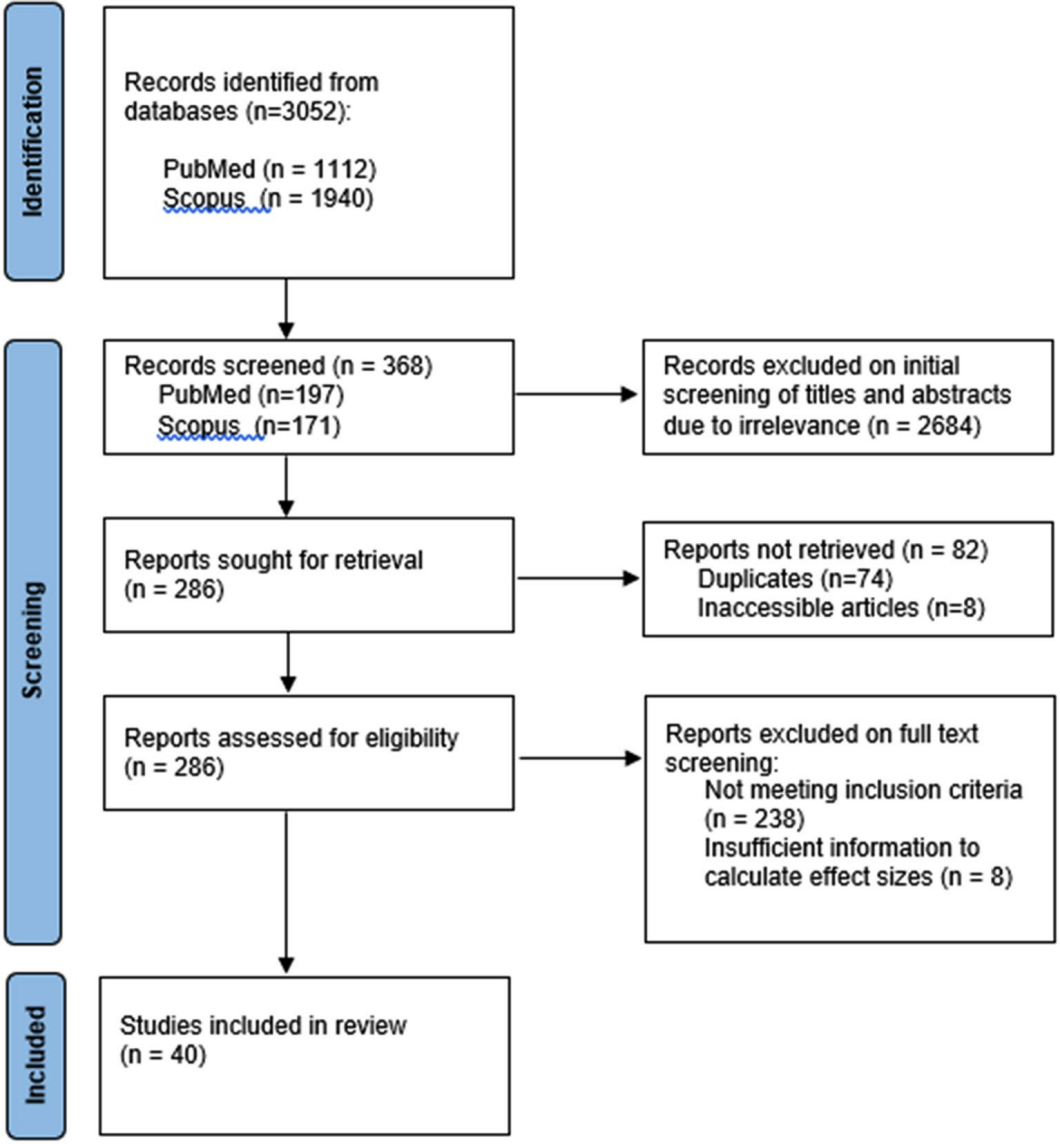
Flowchart of the systematic literature search. The search terms yielded 3052 articles where 2684 were excluded on review of titles and abstracts due to lack of relevance leaving 368 articles. 82 of these articles were not retrieved as 74 were duplicates and 8 were inaccessible in our library or via interlibrary loans. Of the remaining 286 articles, 246 were excluded as 238 did not meet inclusion criteria and 8 had insufficient information to calculate effect sizes despite attempting to contact the corresponding authors. The remaining 40 articles were included for final review.

The search terms yielded 3052 articles. Initial screening of titles and abstracts excluded 2684 articles due to irrelevance, leaving 368 for further screening. Of these articles, 82 were removed as 74 were duplicates and 8 were unavailable in our library and unable to be obtained via interlibrary loans, resulting in 286 articles for review. Of these, 246 articles were excluded as 238 did not meet inclusion criteria and 8 had insufficient information to calculate effect sizes despite attempts to contact the corresponding authors. A majority of studies were excluded as they did not address undergraduate medical student populations, measured subjective measures (e.g., student opinions) or involved research interventions without a control group. In many cases, studies were excluded due to a combination of factors not meeting inclusion criteria. In total, 40 articles were included for final review. A summary of the process is outlined in Fig. 1.

### Study characteristics

**Fig. 2:**
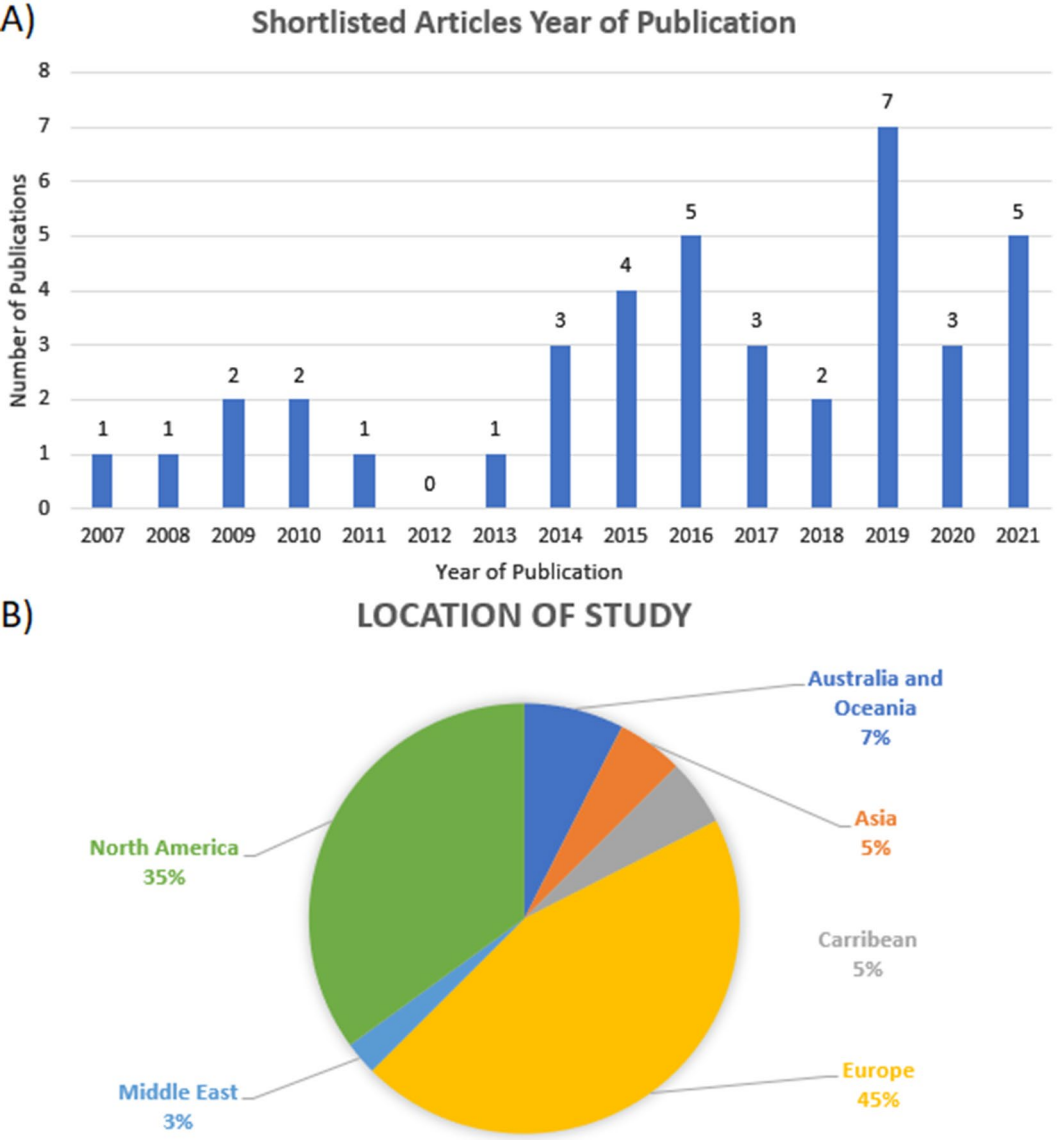
Publication year (**A**) and location of studies (**B**) The majority of the 40 shortlisted studies were published from 2014 onwards (n=32) with the highest number published in 2019 (n=7). Most studies were conducted in Europe (n=18, 45%), followed by North America (n=14, 35%) with USA being the single country with the most studies (n=11, 28%).

From the 40 articles reviewed, 30 consisted of randomised controlled trials (RCTs) and 10 were non-randomised studies. Most were published in 2014 or later, with the greatest number of articles published in 2019 (n = 7, 18%). A large proportion of the studies were conducted in Europe (n = 18, 45%) followed by North America (n = 14, 35%) with USA being the single country with the most studies (n = 11, 28%), see Fig. 2. More studies focused on senior medical students (n = 17, 42.5%), rather than junior medical students (n = 16, 40%). Of the remaining studies, 4 had combined populations of senior and junior medical students (10%) while 3 did not specify the seniority of the cohort (7.5%). The combined studies involved a total of 6242 medical students where population sizes ranged from 17 to 845 (median 101.5; IQR 125.5).

Other extracted study parameters included: active versus passive education delivery; whether eLearning was employed; the imaging modalities taught; and radiology training of the teacher (Supplementary Material 1). Distinctions between the content subgroups of radiologic anatomy, radiation protection and indications for imaging and imaging interpretation were abandoned due to considerable overlap between articles. Many articles did not provide sufficient detail regarding methods of assessment, so that parameter was also omitted from the meta-analysis.

**Fig. 3:**
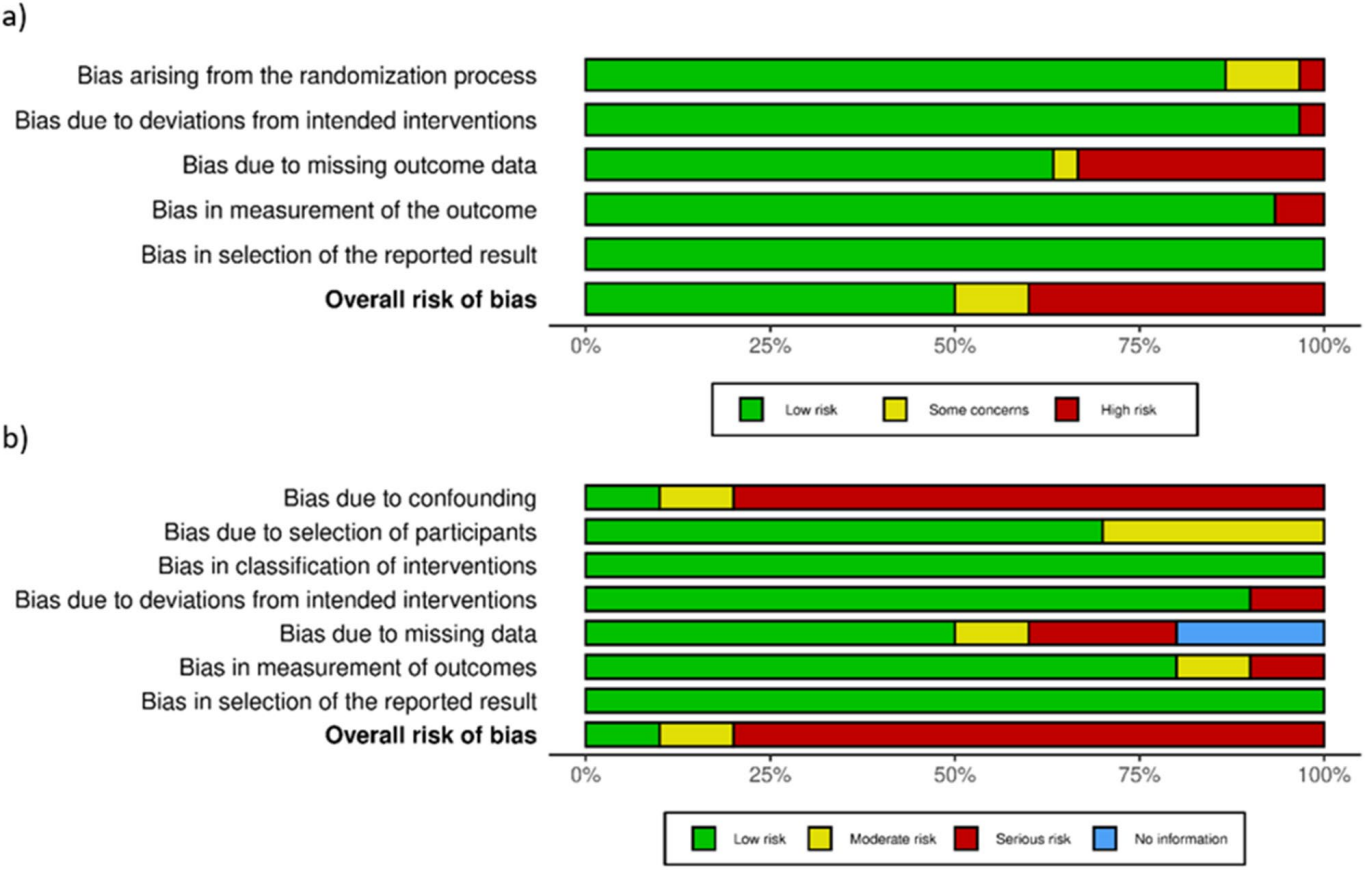
Risk of bias assessment summary for randomised control trials (**A**) and non-randomised trials (**B**)In the randomised trials, there was a serious risk of bias in 12 of 30 randomised studies and moderate risk in 3 of 30 studies. This was predominantly due to missing outcome data and issues from randomisation. In non-randomised trials, 8 of 10 studies were considered at serious risk and 1 study was considered at moderate risk of bias. This was predominantly due to confounding variables followed by missing data and bias in participant selection.

The studies meeting inclusion criteria were generally of good quality with MERSQI scores ranging from 10.5 to 15.5 out of 18, the median score being 13. However, half of the included studies (n = 20) were judged to be at serious risk of bias while 16 were judged to be at low risk of bias (40%) and 4 at moderate risk of bias (10%). Among the included randomised control trials, missing data resulted in a serious risk of bias in 10 of 12 studies and some concerns in 1 study. This was a feature of all three randomized cross over control trials. The main contributor was missing data due to attrition in study groups between phases of these trials. In the non-randomised trials, bias was predominantly due to confounding variables. This featured in all 8 studies deemed at serious risk of bias and contributed to moderate risk in 1 study. A summary of the risk of bias assessments is shown in Fig. 3. A full breakdown of the risk of bias assessment for each included article can be found in Supplementary Material 3 and 4.

**Fig. 4:**
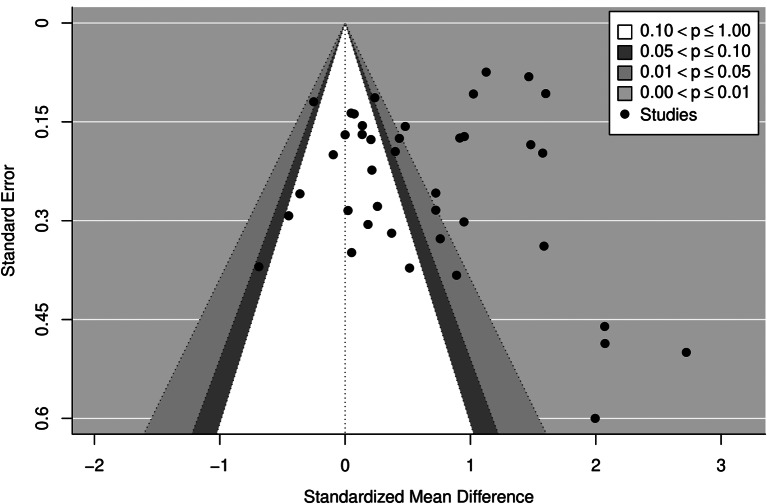
Assessment for publication bias of included studiesThe funnel plot shows the relationship between the effect size and the sample size of the studies included in the systematic review. Studies with high variability and effect sizes near 0 are missing and there are a number of studies which lie outside the expected funnel. In particular it is clear that small studies that have been published are those with relatively large effect sizes (those points on the lower right of the plot). This funnel plot asymmetry suggests publication bias.

A funnel plot analysis demonstrated that studies with high variability and effect sizes near 0 are not present, with multiple studies lying outside the funnel (Fig. 4). In particular, small studies that have been published showed relatively large effect sizes. When overlayed with the p-values of the included studies, only those with p > 0.1 were larger studies. Eggers Test indicated there was evidence of publication bias (Intercept = -0.2841, p < 0.05).A summary of the included articles study characteristics and educational interventions is outlined in Tables 3 and 4 respectively. Brief descriptions of included studies can be found in Supplementary Material 5.

**Table 3:** Characteristics of included studies

					Imaging modalities**		Sample Sizes		Effect Size****
Article	Year of publication	Location	Study Type	Medical School Experience*	Modality	Cross Sectional Imaging***	Intervention	Control	Cohens D
Alamer A. and Alharbi F [12].	2021	Saudi Arabia	RCT	S	N/S	N/S	12	66	1.5867
Beermann J. et al. [40]	2010	Germany	RCT	S	C	Y	103	57	1.4813
Burbridge B. et al. [33]	2015	Canada	Non-randomised	J	M	Y	84	83	0.4796
Courtier J. et al. [75]	2016	US	Non-randomised	S	M	N/S	25	23	-0.4504
Di Salvo D.N. et al. [76]	2014	US	Non-randomised	S	M	Y	23	30	0.2578
Ebert J. and Tutschek B [24].	2019	Switzerland and Germany	RCT	N/S	U	N	21	30	0.0232
van Geel K.V. et al. [47]	2019	Netherlands	RCT	J	X	N	43	60	-0.0947
Gibney B. et al. [77]	2020	Canada	RCT	S	M	Y	26	23	0.9485
James H.K. et al. [34]	2019	UK	RCT	S	R	Y	28	25	0.7234
Knudsen L. et al. [26]	2018	Germany	RCT	J	U	N	22	21	0.1826
Kok E.M. et al. [46]	2015	Netherlands	RCT	J	X	N	61	20	-0.3607
Kok E.M. et al. [78]	2017	Netherlands	RCT	S	X	N	20	20	0.3711
Le C.K. et al. [37]	2019	Canada	RCT	S	U	N	10	7	1.9951
Lorenzo-Alvarez R. et al. [79]	2019	Spain	RCT	J	X	N	53	103	0.1359
Lydon S. et al. [38]	2021	Ireland	RCT	S	M	Y	17	14	2.7221
Mahnken A.H. et al. [32]	2011	Germany	RCT	S	M	Y	32	32	0.7234
Nickel F. et al. [41]	2016	Germany	RCT	S	C	Y	253	157	1.0237
Petersson H. et al. [39]	2009	Sweden	Non-randomised	N/S	M	Y	92	75	0.1377
Poland S. et al. [28]	2018	US	RCT	J	U	N	16	17	0.052
Pusic M.V. et al. [45]	2007	Canada	RCT	S	X	N	69	70	0
Rajprasath R. et al. [35]	2020	India	RCT	J	M	Y	75	75	0.9511
Rozenshtein A. et al. [80]	2016	USA	RCT	J	X	N	20	20	0.759
Saxena V. et al. [36]	2008	USA	Non-randomised	J	M	Y	141	141	-0.2509
Sendra-Portero F. et al. [27]	2013	Spain	Non-randomised	J	N/S	N/S	71	43	0.4001
Shaffer K. et al. [81]	2009	USA	RCT	S	M	Y	38	205	0.206
Smeby S.S. et al. [82]	2019	Norway	RCT	J	M	Y	105	105	0.0723
Stein M.W. et al. [44]	2016	USA	RCT	S	N/S	N/S	100	115	0.0483
Tam M.D.B.S. et al. [30]	2010	UK	RCT	S	C	Y	11	23	0.5142
Thompson M. et al. [83]	2017	USA	RCT	J	X	N	14	15	2.0709
Tshibwabwa E. et al. [21]	2016	Antigua and Barbuda	Non-randomised	J	U	N	378	385	1.4655
Tshibwabwa E. et al. [31]	2017	Antigua and Barbuda	Non-randomised	J	M	Y	473	372	1.1254
Velan G.M. et al. [20]	2015	Australia	RCT	M	M	Y	158	155	0.2368
Viteri Jusue A. et al. [29]	2021	Spain	RCT	S	C	Y	13	13	2.0734
Vollman A. et al. [84]	2014	USA	RCT	M	M	Y	15	16	-0.6884
Wade S.W.T. et al. [23]	2019	Australia	RCT	S	C	Y	63	71	0.4328
Webb A.L. and Choi S [43].	2014	UK	Non-randomised	J	X	N	72	28	0.2137
Weeks J.K. et al. [42]	2021	USA	RCT	J	C	Y	15	15	0.8892
Willis M.H. et al. [25]	2020	USA	Non-randomised	M	M	Y	72	63	1.5765
Wong V. et al. [22]	2015	Australia	RCT	M	M	Y	72	73	0.915
Yuan Q. et al. [85]	2021	China	RCT	N/S	M	Y	232	228	1.6007

^*^Medical school experience– Junior (J), Senior (S), Mixed (M)

^**^Imaging modalities– X-ray (X), Computed Tomography / CT (C), Ultrasound (U), Magnetic resonance imaging (R), Multimodality / Combination (M)

^***^Cross sectional imaging– Yes (Y), No (N)

^****^A positive effect size favours the intervention group while a negative effect size favours the control group

N/S– not specified

**Table 4:** Overview of education delivery methods in included studies

	Educational activity*		eLearing**		Teaching staff ***	Delayed Assessment ****
Article	Intervention	Control	Intervention	Control		
Alamer A. and Alharbi F [12].	C	P	A	N/A	I	N
Beermann J. et al. [40]	C	C	P	P	I	N
Burbridge B. et al. [33]	C	P	A	N/A	I	N
Courtier J. et al. [75]	A	C	A	N/A	I	N
Di Salvo D.N. et al. [76]	C	C	N/A	N/A	I	N
Ebert J. and Tutschek B [24].	A	P	A	P	N/S	Y
Geel K.V. et al. [47]	C	C	P	P	I	N
Gibney B. et al. [77]	P	P	N/A	N/A	I	N
James H.K. et al. [34]	A	A	N/A	N/A	O	N
Knudsen L. et al. [26]	A	C	N/A	N/A	O	Y
Kok E.M. et al. [46]	A	A	P	P	I	N
Kok E.M. et al. [78]	C	P	N/A	N/A	I	N
Le C.K. et al. [37]	A	A	A	N/A	O	N
Lorenzo-Alvarez R. et al. [79]	A	A	A	N/A	I	N
Lydon S. et al. [38]	C	P	N/A	N/A	O	N
Mahnken A.H. et al. [32]	C	P	A	N/A	I	N
Nickel F. et al. [41]	C	C	P	P	N/S	N
Petersson H. et al. [39]	C	P	A	N/A	I	N
Poland S. et al. [28]	A	A	A	N/A	N/S	N
Pusic M.V. et al. [45]	A	A	A	A	N/S	N
Rajprasath R. et al. [35]	C	P	N/A	N/A	O	N
Rozenshtein A. et al. [80]	P	P	P	P	I	N
Saxena V. et al. [36]	C	A	P	N/A	O	N
Sendra-Portero F. et al. [27]	P	P	P	N/A	N/S	N
Shaffer K. et al. [81]	C	C	A	N/A	I	N
Smeby S.S. et al. [82]	A	P	N/A	N/A	I	N
Stein M.W. et al. [44]	A	P	N/A	N/A	I	Y
Tam M.D.B.S. et al. [30]	C	C	P	P	N/S	N
Thompson M. et al. [83]	P	P	N/A	P	I	N
Tshibwabwa E. et al. [21]	A	P	P	P	I	N
Tshibwabwa E. et al. [31]	A	C	A	N/A	I	Y
Velan G.M. et al. [20]	A	P	A	N/A	I	N
Viteri Jusue A. et al. [29]	A	C	A	N/A	N/S	N
Vollman A. et al. [84]	P	P	P	P	I	N
Wade S.W.T. et al. [23]	A	P	A	P	I	N
Webb A.L. and Choi S [43].	C	C	A	N/A	I	N
Weeks J.K. et al. [42]	P	P	P	P	I	N
Willis M.H. et al. [25]	A	P	A	N/A	I	N
Wong V. et al. [22]	A	P	A	P	I	N
Yuan Q. et al. [85]	P	P	P	N/A	N/S	N

^*^Educational Activity– Active (A), Passive (P), Combined (C)

^**^eLearning– Active (A), Passive (P), Not applicable / eLearning not present (N/A).

^***^Teaching staff– Imaging professional (I), Other (O)

^****^Delayed Assessment– Yes (Y), No (N)

N/S– not specified

### Meta-analysis

**Fig. 5:**
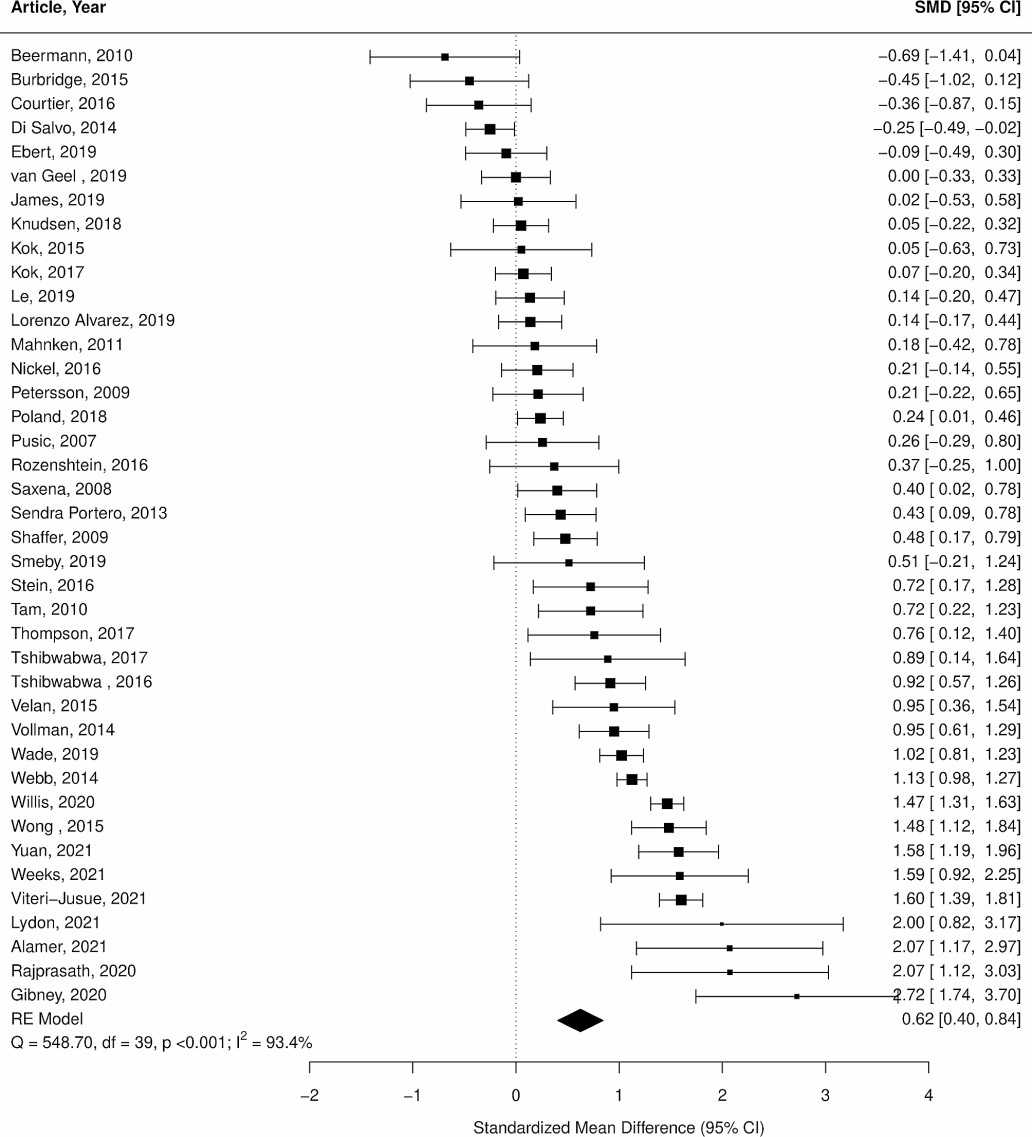
Random effects meta-analysis of studies comparing radiology education interventionsThe majority of education interventions increased students’ radiology knowledge or skills evidenced by a majority demonstrating a shift to the right. However, in general studies with higher standard mean differences had wider confidence intervals. High heterogeneity (I^2^ = 93.4) limited the capacity to draw conclusions from this analysis and a cause was not found in the subgroup analyses).

Considerable heterogeneity between studies (I^2^ = 93.42%) limited the capacity to draw conclusions in this analysis. In subgroup analyses, including comparing eLearning vs. other methods, senior vs. junior medical students, passive vs. active learning, cross-sectional imaging vs. other imaging, radiology-trained vs. non-radiology-trained teaching staff and RCT vs. non-randomised studies, heterogeneity remained high. This suggests none of these were significant contributors to the heterogeneity. A forest plot of the included studies reveals a majority of the educational interventions increased medical students’ radiology knowledge and or skills evidenced by a majority demonstrating a shift to the right. This is displayed in Fig. 5. This is a trend also demonstrated in all forest plots of subgroup analyses which can be found in Supplementary Material 6-11. However, there were no significant differences encountered in the subgroup analyses. It is worthwhile to note a greater proportion studies utilising active learning had a shift to the right in Supplementary Material 6. This resulted in a higher standard mean difference of 0.57 vs. 0.51 however was not statistically significant.

### Thematic analysis

The meta-analysis demonstrated high heterogeneity with no statistically significant differences encountered in the subgroup analyses to account for this. This would suggest educational interventions were highly contextual and thematic analysis was performed to further explore this.

#### Active vs. passive learning

Active learning has been shown to produce superior gains in knowledge acquisition than passive learning [20–25]. In particular, active learning utilising interactive eLearning in several student cohorts demonstrated superior knowledge gains compared with passive methods of instruction [20, 22, 23, 25]. Three of these studies were judged to be at potential serious risk of bias due to missing outcome data. This was as a result of participants dropping out between phases of the study which was likely an inherent risk with all studies involving randomised cross-over control trials [20, 22, 23]. Otherwise these studies were judged to have a low risk of bias in the remaining domains. This attrition could be in part explained by active / interactive learning being associated with greater levels of student satisfaction or intrinsic motivation [22, 23, 26].

#### eLearning vs. face-to-face learning

Multiple studies demonstrated eLearning is at least equivalent to ‘traditional’ face-to-face education [12, 22, 23, 27–30]. Blending eLearning with ‘traditional’ learning pedagogies was reported to have a synergistic effect [31, 32]. Moreover, guided interactive eLearning has been shown to be effective in radiology education and is well accepted by participants [20, 22, 23, 25, 33]. The use of worked examples or clinical scenarios with feedback to demonstrate imaging concepts was effective. However, knowledge gains in these guided eLearning resources appeared to diminish with increasing medical student experience / seniority [22, 23, 33].

#### Specialist vs. non-specialist radiology educators

Most articles employing radiologists as teachers had topics which varied and often overlapped (n = 26, 65%). A majority taught medical imaging indications or interpretation component (n = 21 of 26, 81%). In 6 articles educators were non imaging trained specialists (15%) and in 8 articles instructor training was unspecified (20%). Non-imaging trained specialists were primarily involved in anatomy teaching (n = 3 of 6, 50%), [34–36] followed by ultrasound scanning (n = 2 of 6, 30%) [26, 37] and in one article, interpreting orthopaedic imaging [38]. Considering the meta-analysis, this could suggest a trend toward non-imaging trained teachers being equivalent to imaging trained specialists in teaching basic imaging anatomy and ultrasound scanning. However, there was heterogeneity in the student cohorts and topics taught. This suggests these findings are likely contextual.

#### Medical student seniority

There were 17 studies involving senior medical students, 16 studies involving junior medical students, 4 in a combined group of medical students and 3 were unspecified. Junior students were mostly taught basic imaging interpretation (n = 12/16, 75%), followed by anatomy (n = 8/16, 50%). Imaging as part of anatomy teaching to senior students was relatively less common (n = 7/17, 41%), however more content covering imaging indications was taught to that cohort. Risks and radiation protection were only specified in 4 of 17 studies (24%) involving exclusively senior students and 1 study with a combination of senior and junior students. An overview is provided in Supplementary Material 9.

#### Imaging modalities

Imaging modalities employed were divided into cross sectional imaging (CT and MRI) or non-cross-sectional imaging (x-ray and ultrasound). In 4 studies it was indeterminate whether cross-sectional imaging was taught. Most studies utilised multiple imaging modalities to teach (n = 17/37, 46%) where cross sectional imaging featured in 23 of 36 studies (64%). Cross sectional imaging teaching was employed proportionately more in studies with only senior students (n = 11/14, 79%) compared to studies with only junior students (n = 6/15, 40%).

#### Learning anatomy using imaging

Cross-sectional imaging was frequently used to teach anatomy, however the method in which the anatomy was displayed affects learning [39–41]. 3D representations have been shown to produce significantly superior knowledge gains compared to 2D [39–41]. The use of augmented reality, e.g., 3D CT hologram displays to teach head and neck anatomy, yielded a large effect size when compared with 2D CT images [42]. Using x-rays to teach radiological anatomy yielded only a relatively small effect size in a study 2013 study by Webb and Choi; however, this should be interpreted with caution due to potential bias in this study [43]. In a single study by Knudsen et al. there was no significant difference between the group using ultrasound scanning (hands-on group) and a group which utilized ultrasound images, 3D models and prosections (hands-off group) for learning anatomy [26]. The ultrasound scanning group had significantly higher intrinsic motivation compared to the ‘hands-off’ group which had a greater degree of didactic teaching [26].

#### Indications for imaging

Learning indications for imaging using face-to-face collaborative learning and didactic teaching was equally effective in a cohort of 3rd year medical students [44]. However, collaborative learning was perceived as more enjoyable [44].

eLearning has been successfully used to teach indications for imaging [20, 22, 23, 25, 29]. Engaging interactive eLearning which utilised clinical scenarios and provided feedback, has been showed to produce significantly improved knowledge of imaging indications when compared to non-interactive eLearning [20, 22, 23].

#### Imaging interpretation

Learning how to interpret imaging investigations enabled students to detect suboptimal imaging and to identify abnormalities [45]. However, following an eLearning educational intervention using active learning, students were *less* likely to detect normal imaging compared with abnormal imaging [45]. This could be mitigated by providing comparisons between normal studies and studies showing diseases, as demonstrated by Kok and colleagues [46]. When instruction with the ratio of normal to abnormal studies in imaging sets was varied, there was a trade-off between sensitivity and specificity in imaging interpretation by students [47].

Sequencing of educational interventions also had implications for knowledge acquisition. In groups where expert instruction was provided prior to practice (deductive learning), students demonstrated higher specificity than those who were allowed to practice cases prior to instruction (inductive learning) [47]. The type of learning did not significantly affect sensitivity for detecting pathologies [47].

#### Mandatory vs. voluntary participation

There was a correlation between the number of educational sessions attended and performance on test scores [12, 32]. Overall, students performed significantly better when participation in educational interventions was mandatory [32].

#### Assessment of learning

Most articles did not include a copy of the assessments available and the type or a part of the assessment was not specified in 9 of 40 articles (23%). Delayed testing several months after the educational intervention was only present in 4 of 40 studies (10%). The most common mode of assessment was multiple choice questions (MCQ) followed by short answer questions. Objective Structured Clinical Exams (OSCE) featured in 3 studies, all involving senior medical students. ‘Drag and drop’ or identifying features on imaging was present in one study of junior medical students (6%) and 4 of 17 studies of senior medical students (24%). This suggests that assessments more closely mirroring clinical practice are predominantly used in senior years. An overview is provided in Supplementary Material 5.

## Discussion

There has been increasing interest in undergraduate radiology education, as evidenced by the number of published articles per annum. This review covered a wide variety of educational delivery methods related to several radiology-related topics. This is reflected in the high degree of heterogeneity in the meta-analysis, which did not reduce after subgroup analyses, suggesting these were not significant contributors to heterogeneity.

Educational design aspects addressed in the meta-analysis were active or passive learning and eLearning vs. other forms of delivery. A more granular analysis stratified according to delivery methods such as readings, lectures, flipped or non-flipped classrooms was not possible due to the insufficient number of articles in each sub-category meeting the inclusion criteria for this study. There were many examples in the literature for both medical education in general, and radiology in particular, where active learning has resulted in superior outcomes for knowledge acquisition and/or engagement by participants, compared with didactic approaches [5, 22, 23, 48, 49]. This finding is reinforced by our analysis, where all articles directly comparing the outcomes of active versus passive approaches had effect sizes favouring active learning. Likewise, all studies which evaluated combined active and passive approaches versus passive learning only had effect sizes favouring groups utilizing active learning. However, there was no significant difference between groups exposed to active learning versus passive learning methods in subgroup analyses. This finding could be related to the confounding effect of studies which compared a combination of active and passive approaches with passive learning.

Another factor impacting these findings is the instructional design of educational interventions. In eLearning, for example, effective strategies included use of multimedia learning principles, i.e., relevant graphics to accompany text, arrows to direct attention in complex graphics (signalling principle), using simple graphics to promote understanding while avoiding irrelevant information to maintain coherence and breaking down topics into small logical segments [50]. Teaching of imaging concepts prior to practical applications such as worked examples which fade to full practice scenarios accompanied by feedback is also effective [50]. These principles can all be integrated into teaching anatomy or basic imaging interpretation, the two most commonly addressed topics by articles included in this study.

There are conflicting findings in the literature comparing the efficacy of eLearning versus face-to-face learning in healthcare education [51–57]. This study demonstrated that eLearning is at least equivalent to traditional face-to-face instruction and may be synergistic with face-to-face teaching. However, several forms of guided eLearning in this study appear to have diminishing effects with increasing medical student experience / seniority.^59^ In this scenario, worked examples could impede learning in more experienced participants through the ‘expertise reversal effect.’ [58] Gradually fading ‘worked examples’ into ‘practice questions’ could overcome this concern [58]. These findings are particularly relevant with the massive expansion of eLearning in medical education, including radiology, during the COVID-19 pandemic. Furthermore, there are ongoing barriers to engaging radiologists in education of medical students due to competing clinical demands, thereby increasing the attractiveness of employing eLearning for radiology education [59]. In designing an eLearning intervention, interactivity, practice exercises, repetition and feedback have been shown to improve learning outcomes [22, 23, 52, 60].

This study included articles demonstrating synergies can be achieved between radiology education and the broader medical curriculum [21–23, 33]. For example, there are many instances where cross sectional imaging has been used to teach anatomy [39, 61–63].

Instructional design of e-learning materials influences learning. An example of this includes the use of ‘worked examples’ in e-learning tutorials which were designed for a cohort of senior medical students [23]. This format highlighted relevant clinical information which likely contributed to greater learning efficiency though greater mean scores and/or less time spent interacting with resources by the intervention group [22, 23, 58]. According to cognitive load theory, cognitive overload can occur when information exceeds the learner’s capacity for processing information in their working memory [22, 23, 58, 64]. The result is incomplete or disorganised information [22, 23, 58, 64]. The way information is presented can influence extraneous load imposed by instructional design [64]. To avoid cognitive overload in these e-learning modules, information was concise, pitched at the level of the learner and appropriately segmented [22, 23, 58, 64]. Participants favoured the concise, case-based nature of the tutorials which promoted interactivity and engagement [23, 58]. These studies provide evidence to suggest that students’ learning would benefit from greater integration of radiology into modern medical curricula in a way which is relevant to clinical practice.

### Implications for radiology education and study design

The implications of this review for design of interventions and evaluative studies of radiology education are summarised in Table 5.

**Table 5:** Principles and recommendations, from a systematic review and meta-analysis on undergraduate radiology education

Principles	Notes	Recommendation
**Study design**		
Comparison of the intervention with a control group.	Many excluded articles encountered educational research without a control. These ‘justification studies’ usually yield large effect sizes and are not necessarily informative on efficacy of an intervention relative to an existing program [86].	Studies comparing educational interventions should include direct comparison between a control and intervention cohort. Ideally, pre- and post-test should be conducted for both groups and post-test results adjusted or randomisation stratified for baseline differences.
Evaluation of quantitative knowledge / skill assessment, rather than subjective perceived gains in knowledge.	Many excluded articles involved qualitative analysis without quantitative analysis of knowledge or skill acquisition. Perceptions can differ from objectively measured attainment of knowledge or skills [65, 66].	Where research question includes assessment of knowledge / skills gains, quantitative analysis of knowledge and / or skill acquisition yields should be conducted instead of assessment of participants opinion on knowledge / skills they gained.
Definitions of experimental and control group educational interventions.	Ambiguity in descriptions of educational interventions can limit accurate comparison and reproducibility. Often control groups were reported in less detail.	Detailed description of both educational interventions and control treatment should be reported. As a minimum, this should include student cohort, studied topics, methods of delivery and teaching time.
Immediate post-intervention versus delayed testing.	Knowledge fades over time [87–89] and delayed testing could inform on the degree of reinforcement required to maintain knowledge for future clinical practice. Only a small number of studies described delayed testing.	Studies using short and long-term knowledge retention testing should be conducted when evaluating medical student radiology education programs.
Reporting of studies with negative results (publication bias).	There was evidence of publication bias where small studies showed relatively large effect sizes.	Methodologically sound research should be published regardless of the outcome being positive or negative. Alternatively, researchers should consider using state of the art statistically principled bias correction methods [90].
**Design of educational interventions **		
Heterogeneity in radiology education interventions and examinations.	Exposure to radiology teaching in medical schools and subsequent medical students’ imaging knowledge varies. Methods of assessment also vary as demonstrated in this study. This heterogeneity could confound study results.	Suggested radiology curricula exist [91, 92] and greater adoption of these could reduce heterogeneity for future studies. Adoption of standardised medical student radiology examinations with validated questions could also help drive more uniform curriculum development and its evaluation [93].
Thematic analysis suggests synergisms exist between radiology and anatomy education.	Cross-sectional imaging, including CT and ultrasound have been used to teach anatomy. 3D representations, when possible, may be superior compared to 2D stacks of images.	Anatomy and imaging education can be synergistic. However, the method of displaying anatomy may impact educational effectiveness. More studies are needed to investigate this.
Thematic analysis suggests active learning could produce superior gains in knowledge or skill acquisition compared to passive learning.	All studies directly comparing knowledge or skill acquisition in active learning versus passive learning had effect sizes favouring active learning. Interactivity with active learning was associated with greater student satisfaction.	Passive or didactic learning could be used to introduce theory. Active learning should be used to revise and apply theory. Examples include imaging selection or interpretation.
eLearning is equivalent to traditional face to face education.	eLearning can be synergistic with traditional teaching. However, its effectiveness varies with instructional design. Thematic analysis suggests active learning or methods utilising guided teaching are associated with higher effectiveness.	eLearning should contain an interactive component and be produced in keeping with the best principles of instructional design. Examples include ‘worked examples’ or practice questions with feedback.

### Study strengths

To the authors knowledge, this is the first systematic review and meta-analysis aimed at quantitively comparing the effectiveness of different methods of radiology education for medical students. This review captured a large quantity of articles and a large medical student population dating back 15 years.

Through the application of stringent search criteria, only comparative effectiveness studies which were generally of high quality were shortlisted, as evidenced by high MERSQI scores. This study also excluded qualitative studies assessing perceived gains in knowledge or skills, because perceptions can differ from objectively measured attainment of knowledge or skills [65, 66].

### Study limitations

The main limitation of this study is the high level of heterogeneity between studies. Significant heterogeneity existed between the shortlisted articles regarding the topics studied, study methods, data collection and reporting. This is unsurprising, because medical curricula vary widely, and educational interventions are typically contextual [3–5]. The interventions in many cases were designed for specific populations to address specific educational needs related to radiology. The high heterogeneity in this meta-analysis has also been demonstrated in other medical and health sciences-related meta-analyses of educational effectiveness [60, 67, 68].

Descriptions of interventions and reporting of data in some studies were ambiguous which complicated data extraction. This more commonly occurred in descriptions of control groups. Frequently, critical aspects of studies were reported in insufficient detail, which has been encountered in other reviews [52, 57, 60, 68]. While the authors tried to ensure categorisation was as accurate as possible, in some instances their ability to do so was limited due to the ambiguities in reporting of the data.

Moderate to high levels of bias and evidence of publication bias in the shortlisted articles is another limitation which impacts on the ability to draw conclusions from the meta-analysis. This suggests published literature may be skewed towards studies reporting effectiveness of the interventions and negative results being potentially under-reported. Prevalent sources of bias such as missing data and confounding variables highlight the need to be vigilant when evaluating education interventions. This paper is limited to peer-reviewed articles in the PubMed and Scopus databases. Articles identified in the reference lists of included articles, as well as grey literature and unpublished sources were not included. This restriction was intended to maintain the reliability of this review’s method.

### Future directions

The authors recommend better standardisation of the design of studies examining educational interventions in general, and radiology in particular, to help determine the most effective methods for teaching undergraduate medical students. Greater use of delayed testing to evaluate long term effectiveness of educational interventions is needed. This could inform educators regarding the reinforcement required to maintain knowledge for future clinical practice.

Further research is needed to analyse the effectiveness of integration of radiology education with other disciplines in medical curricula. It stands to reason that integration of radiology with basic sciences and clinical experiences would lead to synergistic benefits for students’ learning. Disciplines such as anatomy, pathology, and clinical reasoning might all benefit from integration with radiology [2, 9, 69–74]. When combined with delayed testing, this could inform curriculum planners on when to incorporate radiology topics into medical curricula.

## Conclusion

There has been increasing research interest in radiology education for medical students. However, methods of educational delivery and evaluation vary widely, thus contributing to significant heterogeneity between studies. A comprehensive subgroup analysis did not reveal a cause for this heterogeneity, suggesting that it could be due to tailoring of educational interventions for specific curricular contexts.

While heterogeneity precluded any firm conclusions being drawn from the meta-analysis, this systematic review has explored scenarios where certain educational interventions and specific improvements in future study design can be of benefit. For example, eLearning has been shown to be at least equivalent to traditional face-to-face instruction and may be synergistic. Better standardisation in the design of studies to evaluate radiology education interventions and in the nature of the radiology education interventions themselves is needed to help provide evidence for the optimization of radiology education in medical curricula. Other potential research directions might include evaluating long-term knowledge retention through delayed testing of learning as well as further work to demonstrate the effect of integrating radiology education with other disciplines within medical curricula.

### Electronic supplementary material

Below is the link to the electronic supplementary material.

Supplementary Material 1: Data Points Extracted from Shortlisted Articles

Supplementary Material 2: Definitions of Data Points

Supplementary Material 3: Risk of Bias Assessment for Randomised Trials

Supplementary Material 4: Risk of Bias Assessment for Non-Randomised Trials

Supplementary Material 5: Topics and Modes of Assessment According to Student Seniority

Supplementary Material 6: Active versus Passive Learning Subgroup Analysis

Supplementary Material 7: eLearning versus No eLearning Subgroup Analysis

Supplementary Material 8: Cross-Sectional Imaging versus No Cross-Sectional Imaging Subgroup Analysis

Supplementary Material 9: Student Seniority Subgroup Analysis

Supplementary Material 10: Imaging Professionals versus Non-Imaging Professionals as Teachers Subgroup Analysis

Supplementary Material 11: Study Design Subgroup Analysis

## Data Availability

The datasets used and/or analysed during the current study are available from the corresponding author on reasonable request.

## Abbreviations

CT: Computed Tomography
DSA: Digital Subtraction Angiography
MERSQI: Medical Education Research Study Quality Instrument
MCQ: Multiple Choice Question
MRI: Magnetic Resonance Imaging
OSCE: Objective Structure Clinical Exams
PBL: Problem Based Learning
PRISMA: Preferred Reporting Items for Systematic Reviews and Meta-Analyses
RCT: Randomised controlled trial
SMD: Standard Mean Difference
US: Ultrasound
VIVA: Viva voce / oral examination
