# Supplementary material for: What works in radiology education for medical students: a systematic review and meta-analysis
Source: BMC Med Educ. 2024 Jan 10;24:51. doi: 10.1186/s12909-023-04981-z (PMC10782640; doi:10.1186/s12909-023-04981-z)
Supplement: Supplementary file 1 — Supplementary Material 1: Data Points Extracted from Shortlisted Articles [file 12909_2023_4981_MOESM1_ESM.docx]

Appendix 1 - Data Points Extracted from Shortlisted Articles

1. **Publication details** – country and year of publication
2. **Study Type** – randomised vs non randomised trials
3. **Medical student year of training –** junior medical students defined as though less than halfway through their undergraduate medical course while senior medical students are those at or more than halfway through their undergraduate medical course.
4. **Educational delivery**
   1. **Active versus Passive Learning**
   2. **E-learning** – including active vs passive eLearning.
   3. **Non-e-Learning** – lectures, reading, workshop, problem-based learning (PBL), bedside or as part of a clerkship.
   4. **Instructor** – imaging professional as radiologists (including radiology trainees), sonographers and radiographers or non-imaging professionals.
5. **Content**
   1. **Imaging modalities** – cross sectional imaging as computed tomography (CT) and magnetic resonance imaging (MRI) or non-cross-sectional imaging as x-ray and ultrasound.
   2. ***Radiologic anatomy**
   3. ***Radiation protection and indications for imaging.**
   4. ***Imaging Interpretation.**
6. ***Method of Assessment.**
   1. **Theory** – multiple choice, short answer or combined.
   2. **Practical** – multiple choice, short answer (including report writing), OSCE (including ultrasound scanning or VIVA) or combined.
   3. **Combined theoretical and practical components.**
7. **Effect size information** – effect sizes (if quoted) or information to calculate effect sizes (as mean and standard deviation).

**Notes:**

- *This information was intended to be included in the original study design. However, it could not be obtained from the shortlisted articles due to consistently limited or ambiguous description. This precluded the final comparison of these aspects of educational design / delivery.
- Lectures also included multi-disciplinary team meetings or case conferences.
