# Supplementary material for: What works in radiology education for medical students: a systematic review and meta-analysis
Source: BMC Med Educ. 2024 Jan 10;24:51. doi: 10.1186/s12909-023-04981-z (PMC10782640; doi:10.1186/s12909-023-04981-z)
Supplement: Supplementary file 2 — Supplementary Material 2: Definitions of Data Points [file 12909_2023_4981_MOESM2_ESM.docx]

Appendix 2 - Definitions of Data Points

**Year of Publication –** the year in which the paper was published. **Location –** the country in which the research took place. **Medical student experience –** students were classified as junior if they were less than halfway through their undergraduate medical course or senior if they were more than halfway through their undergraduate medical course. **Active learning** – ‘two-way’ transmission of knowledge such as problem-based learning, interactive eLearning or practical classes.

**Passive learning** – ‘one-way’ transmission of knowledge such as didactic lectures, reading and or presentations.

**eLearning** – primary stand-alone web-based or electronic (e.g., CD) form of education delivery such as videos, an interactive program or stand-alone PowerPoint presentation. If used as an adjunct to non-web-based / electronic delivery, for example a PowerPoint presentation as part of a lecture, this was not considered eLearning for the purposes of data analysis.

**Imaging professional** – for the purposes of this review is defined as educators who have or are training in medical imaging and include radiologists, radiology trainees, sonographers and radiographers.

**Non imaging professional** – for the purposes of this review is defined as educators whose primary training is not in medical imaging. This can include medical specialists or doctors in training from other specialties (such as emergency medicine) or university academics with expertise in areas other than radiology (e.g., anatomy).

**Modality** – the imaging technique(s) used in the education program which include cross-sectional imaging as computed tomography (CT) and magnetic resonance imaging (MRI), non-cross-sectional imaging as x-ray and ultrasound (US) or a combination. Fluoroscopy and digital subtraction angiography (DSA) were incorporated into x-ray as these produced planar imaging similar to x-ray.

**Cross sectional imaging** – is defined as imaging producing a series of images or stacks demonstrating anatomy in cross section. These are typically shown in standardised planes as axial, coronal and sagittal whose data sets can be manipulated or reconstructed to emphasise anatomy for example CT and MRI. Ultrasound for the purposes of this review was not defined as cross sectional imaging and instead was placed in a separate category, as scan planes and visualisation of anatomy can vary with operator technique and experience.

**Radiologic anatomy** – education which focused primarily on teaching anatomy and its relationships with medical imaging.

**Radiation protection and indications for imaging** – education which focused on appropriate indications for imaging including contraindications, benefits and risks (such as radiation exposure).

**Imaging interpretation** – education which focussed on how to interpret imaging, differentiate abnormal from normal imaging and define pathology.
