# Supplementary figures and images for: What works in radiology education for medical students: a systematic review and meta-analysis
Source: BMC Med Educ. 2024 Jan 10;24:51. doi: 10.1186/s12909-023-04981-z (PMC10782640; doi:10.1186/s12909-023-04981-z)

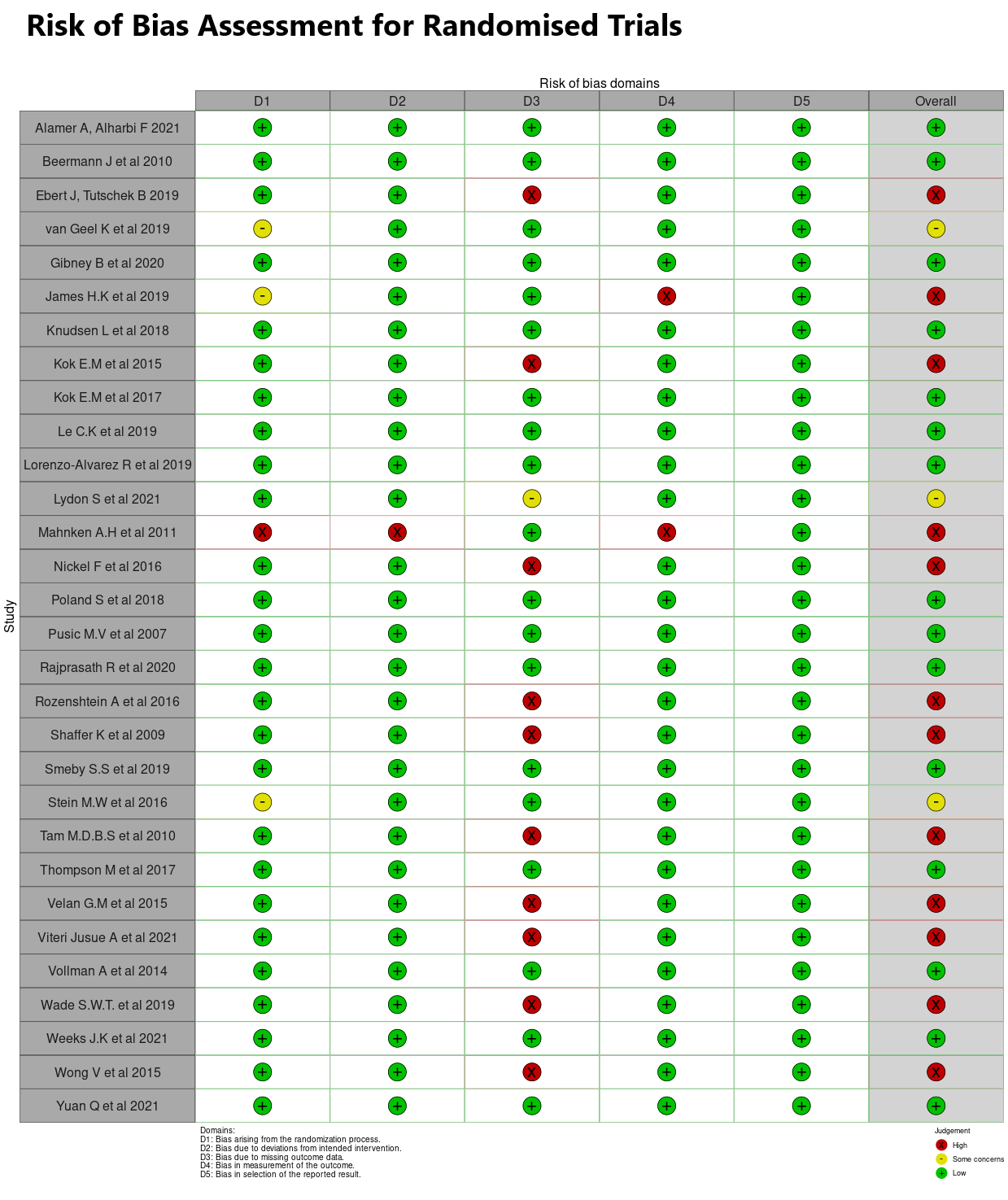

Supplement: Supplementary file 3 — Supplementary Material 3: Risk of Bias Assessment for Randomised Trials [file 12909_2023_4981_MOESM3_ESM.tif]

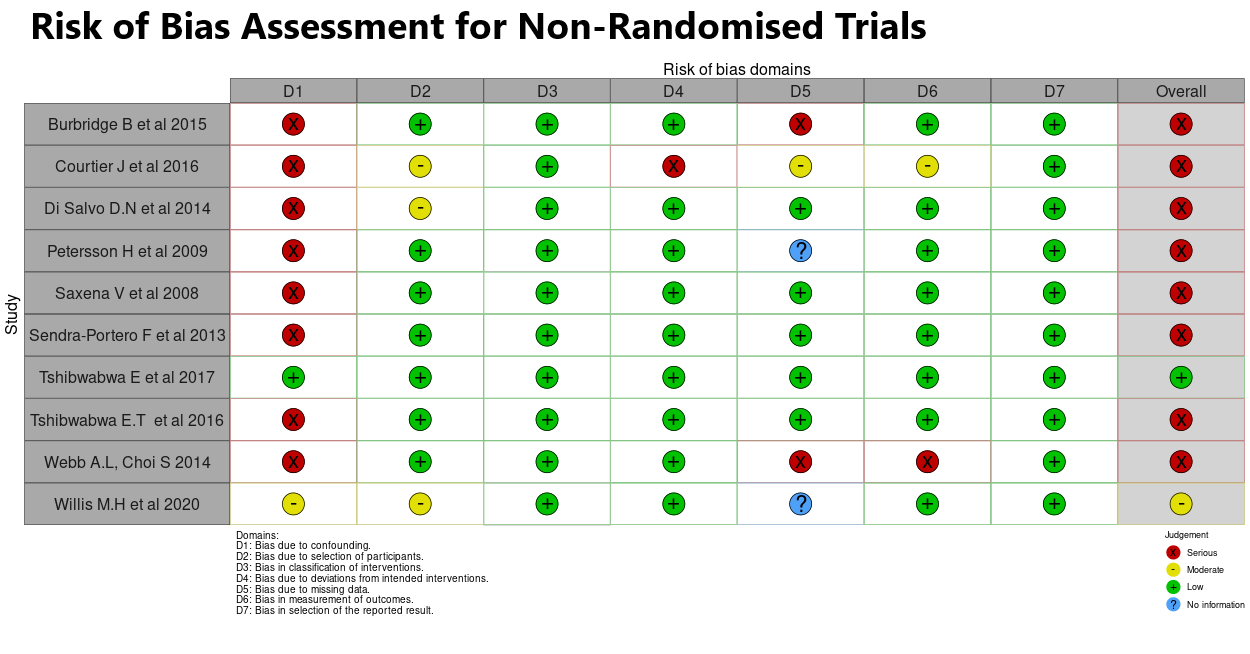

Supplement: Supplementary file 4 — Supplementary Material 4: Risk of Bias Assessment for Non-Randomised Trials [file 12909_2023_4981_MOESM4_ESM.tif]

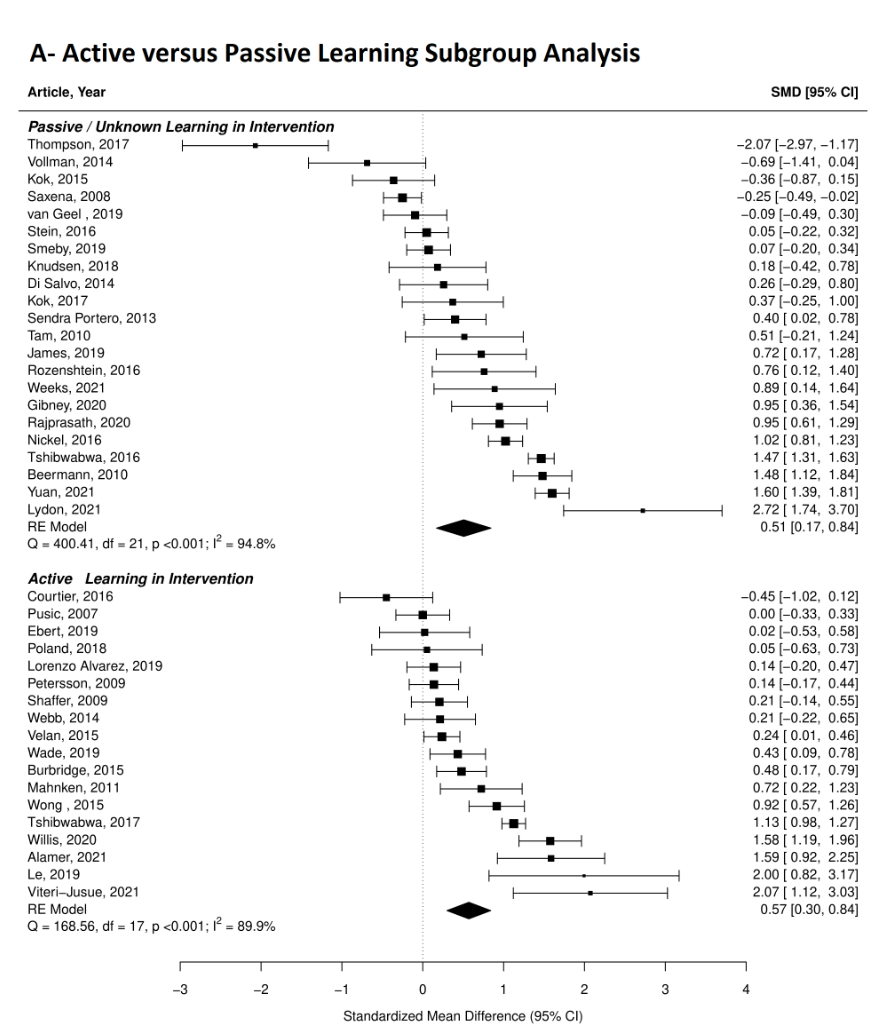

Supplement: Supplementary file 6 — Supplementary Material 6: Active versus Passive Learning Subgroup Analysis [file 12909_2023_4981_MOESM6_ESM.tif]

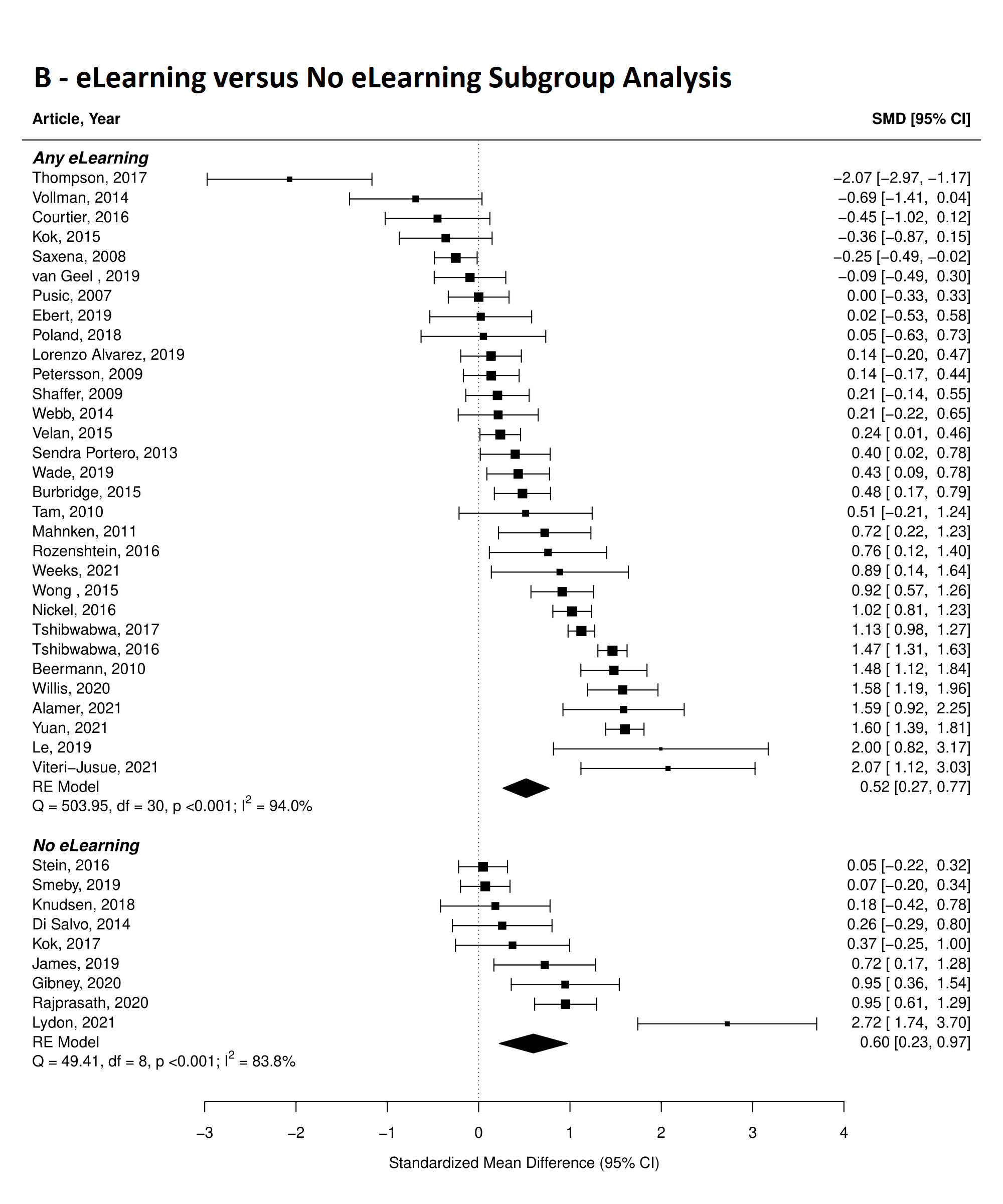

Supplement: Supplementary file 7 — Supplementary Material 7: eLearning versus No eLearning Subgroup Analysis [file 12909_2023_4981_MOESM7_ESM.tif]

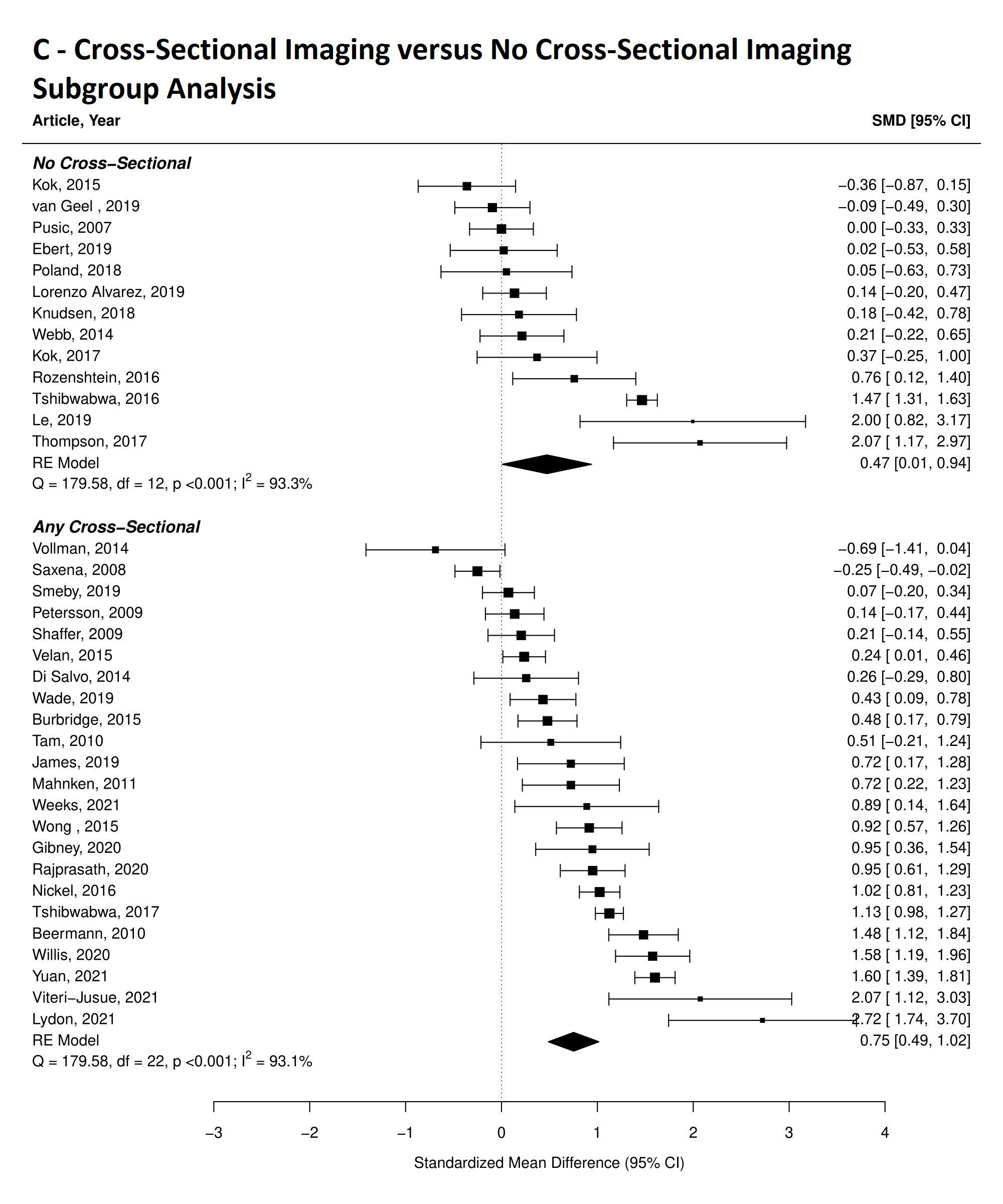

Supplement: Supplementary file 8 — Supplementary Material 8: Cross-Sectional Imaging versus No Cross-Sectional Imaging Subgroup Analysis [file 12909_2023_4981_MOESM8_ESM.tif]

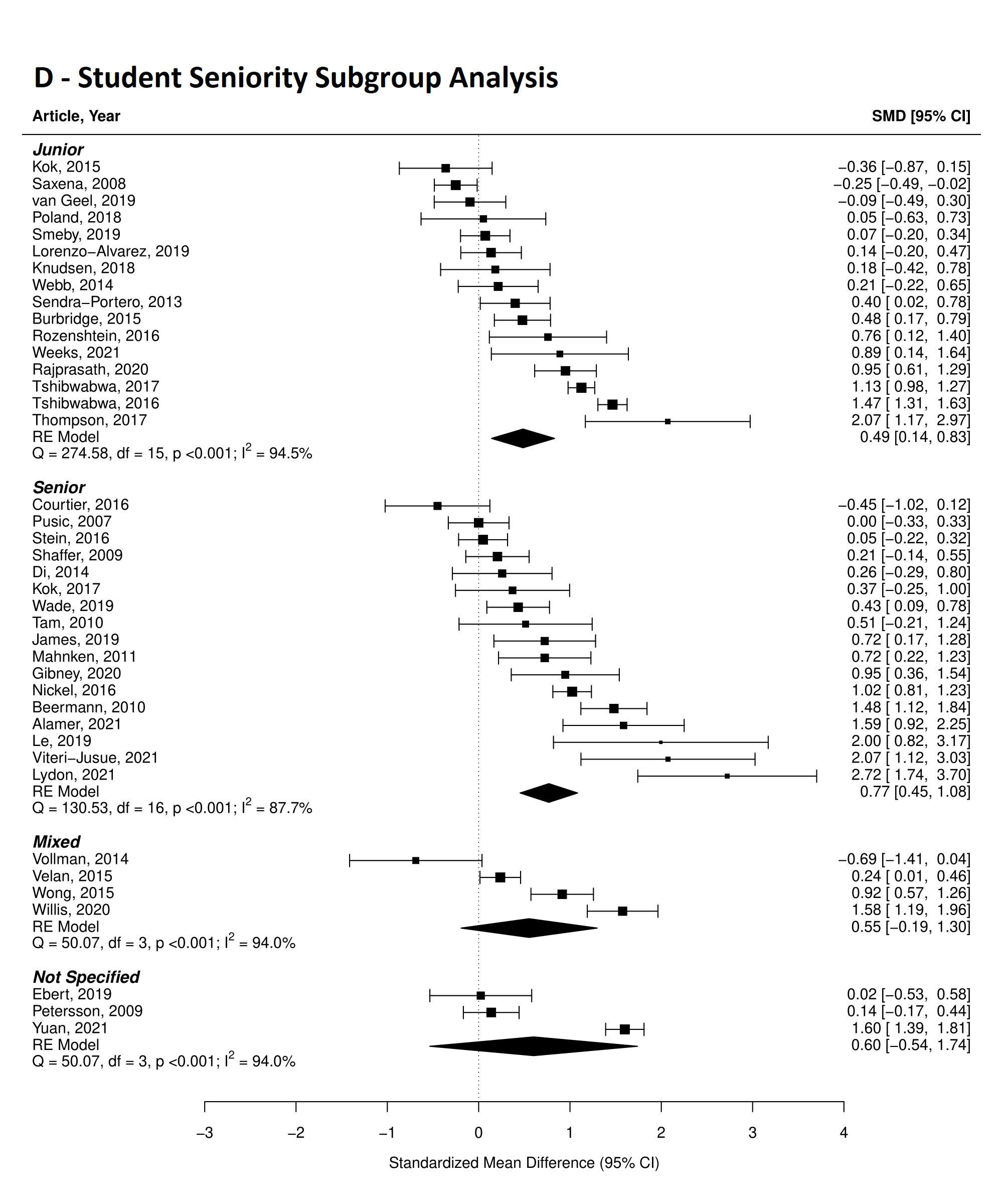

Supplement: Supplementary file 9 — Supplementary Material 9: Student Seniority Subgroup Analysis [file 12909_2023_4981_MOESM9_ESM.tif]

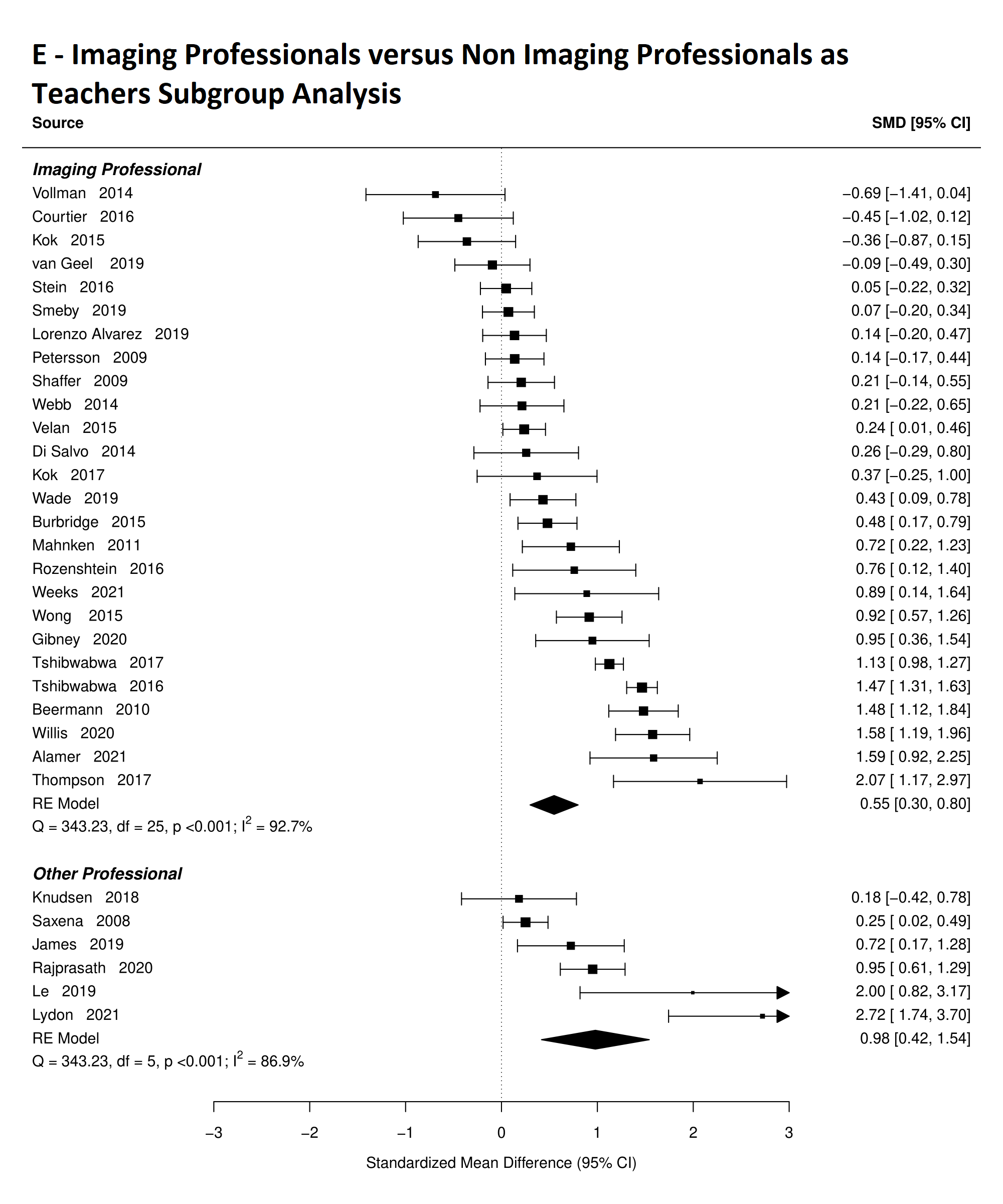

Supplement: Supplementary file 10 — Supplementary Material 10: Imaging Professionals versus Non-Imaging Professionals as Teachers Subgroup Analysis [file 12909_2023_4981_MOESM10_ESM.tif]

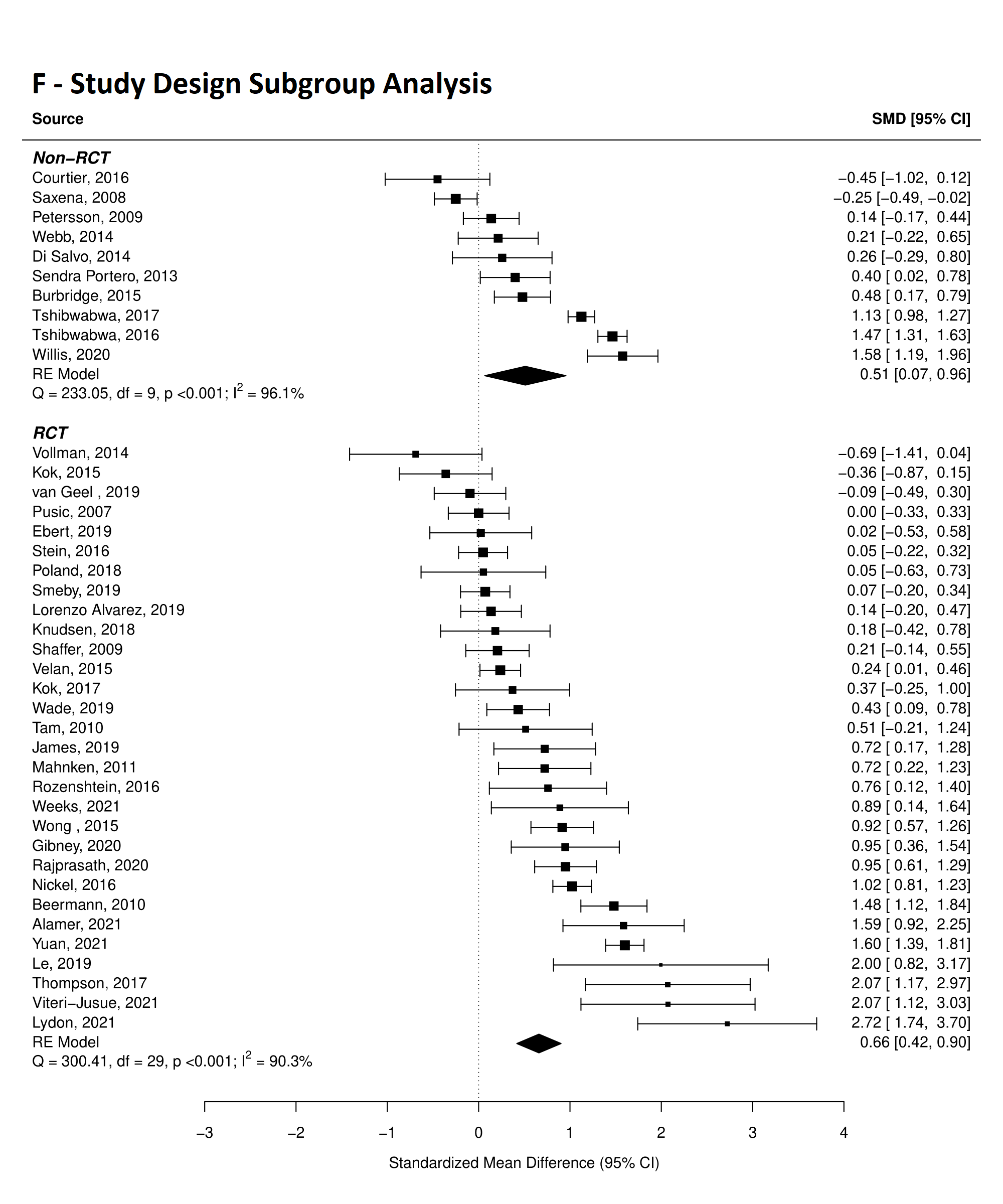

Supplement: Supplementary file 11 — Supplementary Material 11: Study Design Subgroup Analysis [file 12909_2023_4981_MOESM11_ESM.tif]
