# Supplementary material for: What works in radiology education for medical students: a systematic review and meta-analysis
Source: BMC Med Educ. 2024 Jan 10;24:51. doi: 10.1186/s12909-023-04981-z (PMC10782640; doi:10.1186/s12909-023-04981-z)
Supplement: Supplementary file 5 — Supplementary Material 5: Topics and Modes of Assessment According to Student Seniority [file 12909_2023_4981_MOESM5_ESM.docx]

Appendix 5 – Topics and Modes of Assessment According to Student Seniority

| **ARTICLE** | **TOPICS** | | **ASSESSMENT** |
| --- | --- | --- | --- |
| ***Junior Medical Students (n= 16)*** | | | |
| Burbridge B. et al.^34^ | Teaching 1^st^ year students’ anatomy and basic imaging interpretation. Comparison of using eLearning University of Saskatchewan Radiology Courseware versus pre-existing teaching. | An, Bi | MCQ |
| Geel K.V. et al.^48^ | 3^rd^ year student receiving teaching on chest x-ray interpretation. Comparison of using 30% normal versus 70% normal x-rays for teaching. | Bi | MCQ, SA |
| Knudsen L. et al.^27^ | 1^st^ year students receiving ultrasound anatomy interpretation. Comparison using scanning (hands on) or no hands-on training. | An, Us | MCQ, SA (MRT) |
| Kok E.M. et al.^47^ | 3^rd^ year students using case comparisons to interpret chest x-rays. Comparison of using normal versus same condition versus different conditions in teaching. | Bi | MCQ, DD |
| Lorenzo-Alvarez R. et al.^81^ | 3^rd^ year students receiving abdominal x-ray interpretation teaching. Comparison of using an eLearning virtual classroom versus in person teaching. | Bi | MCQ |
| Poland S. et al.^29^ | 3^rd^ and 4^th^ year students learning ultrasound scanning during a practical. Comparison of synchronous eLearning vs in person teaching. | Bi, Us | MCQ, US |
| Rajprasath R. et al.^36^ | 1^st^ year students receiving imaging anatomy teaching with x-rays and CT. Comparison of an integrated radiological anatomy module vs traditional small group teaching. | An | N/S |
| Rozenshtein A. et al.^82^ | Teaching 1^st^ and 2^nd^ year student’s chest x-ray interpretation. Comparison of massed versus interleaved eLearning. | Bi | N/S |
| Saxena V. et al.^37^ | Teaching 1^st^ year medical students’ anatomy using radiological correlation. Comparison of using instructional anatomy videos versus teaching without instructional anatomy videos. | An | MCQ, SA |
| Sendra-Portero F. et al.^28^ | Teaching 3^rd^ year students’ general radiology in a short course of 30 lessons. Comparison of an asynchronous online lecture or in person teaching. | An, In, Bi | OR, SA |
| Smeby S.S. et al.^84^ | Teaching 3^rd^ year students’ neuroradiology with basic imaging interpretation on CT and MRI. Comparison of using express team-based learning versus a traditional lecture. | Bi | MCQ, SA |
| Thompson M. et al.^85^ | Teaching 2^nd^ year student’s lateral chest x-ray interpretation. Comparison of using a mnemonic versus self-study using eLearning (websites and teaching files). | Bi | N/S (Section of ‘documenting evidence of 6 pathologies’). |
| Tshibwabwa E. et al.^22^ | Teaching 1^st^ year students US interpretation via a 2-hour US session. Comparison of small group problem-based learning (PBL) versus large group teaching. | An, Bi, Us | MCQ |
| Tshibwabwa E. et al.^32^ | Teaching 2^nd^ year students’ basics in radiology in two 2-hour sessions. Comparison of combined face to face teaching with eLearning versus face-to-face teaching only. | In, Bi | MCQ |
| Webb A.L. and Choi S.^44^ | 1^st^ year students receiving interactive radiological anatomy and basic x-ray interpretation. Comparison of eLearning (technology enhanced learning and teaching / TELT solution) versus traditional teaching only. | An, Bi | MCQ, N/S (Section on ‘summative course assessment’). |
| Weeks J.K. et al.^43^ | 1^st^ year students receiving head and neck anatomy teaching using CT images. Comparison of using augmented reality versus a conventional computer screen for displaying images. | An | MCQ |
| ***Senior Medical Students (n=17)*** | | | |
| Alamer A. and Alharbi F.^13^ | Radiology clerkship teaching 4^th^ year students imaging indications, basic interpretation, risks, and radiation protection. Comparison of in-person (pre-COVID) and eLearning +/- in-person (post COVID). | In, Bi, Rrp | MCQ, OR |
| Beerman J., et al.^41^ | 4^th^ and 5^th^ year students’ given a teaching module on liver anatomy using CT images. Comparison of 2D stacks vs 3D reconstructions. | An | MCQ, SA, DD |
| Courtier J. et al.^77^ | 4^th^ year students given a paediatric gastoenterology teaching module in basic interpretation while on radiology elective. Comparison of interactive digital game vs interactive style lecture. | In | N/S  (Section specifying ‘end of rotation exam’). |
| Di Salvo D.N. et al.^78^ | 3^rd^ year students’ radiology teaching clerkship at Harvard University teaching imaging indications, anatomy, basic interpretation, risks, and radiation protection. Comparison of traditional clerkship versus revised clerkship. | An, In, Bi, Rrp | OSCE |
| Gibney B. et al.^79^ | Final year students given a lecture on basic image interpretation while on a 1-week radiology clerkship. Comparison of using metaphoric signs versus comparing to normal anatomy during an imaging interpretation lecture. | Bi | SA |
| James H.K. et al. ^35^ | 3^rd^ and 4^th^ year students receiving an anatomy teaching session with radiological correlation. Comparison of using sagittal plastinated slices versus traditional anatomy teaching methods. | An | SA |
| Kok E.M. et al.^80^ | 4^th,^ 5^th^ and 6^th^-year students receiving teaching on chest x-ray interpretation. Comparison of using a checklist versus no checklist. | Bi | SA |
| Le C.K. et al.^38^ | 4^th^ year students receiving ultrasound training while on a 4-week emergency medicine rotation. Comparison of using a simulator versus no simulator as part of an ultrasound teaching program. | An, Bi, Us | MCQ, SC |
| Lydon S. et al.^39^ | 3^rd^ year students receiving teaching on musculoskeletal radiology basic imaging interpretation. Comparison of Say-All-Fast-Minute-Everyday-Shuffled (SAFMEDS) versus a didactic lecture in teaching musculoskeletal radiology interpretation. | Bi | SA |
| Mahnken A.H. et al.^33^ | Comparison of eLearning versus traditional in person vs blended radiology teaching in a 4^th^ year student cohort on a 1-week radiology clerkship. | Bi | N/S |
| Nickel F. et al.^42^ | 3^rd^ to 5^th^ year students using eLearning to learn surgical liver anatomy using CT. Comparison of using 2D imaging stacks versus 3D reconstructions. | An | MCQ, SA, DD |
| Pusic M.V. et al.^46^ | Teaching final year students cervical spine x-ray interpretation. Comparison linear vs web-style eLearning layouts. | Bi | MCQ, DD |
| Shaffer K. et al.^83^ | 3^rd^ year medical student yearlong curriculum in imaging focusing on ambulatory and multidisciplinary medicine as part of an integrated clerkship. Comparison of a new integrated curriculum versus traditional curriculum in a radiology clerkship. | In, Bi, Rrp | MCQ, OSCE, N/S (Section specifying ‘imaging exams’). |
| Stein M.W. et al.^45^ | Teaching 3^rd^ year students imaging indications while on radiology clerkship. Comparison of didactic versus group based / collaborative learning. | In | MCQ |
| Tam M.D.B.S et al.^31^ 2010. | 3^rd^ year students using radiology to learn anatomy via eLearning. Comparison of guided versus unguided access to an eLearning program. | An | SA, N/S (testing factual knowledge and labelling) |
| Viteri Jusue A. et al.^30^ | 6^th^ year medical students learning indications for imaging and making clinical decision based on radiology reports. Comparison of eLearning versus in person teaching. | In | SA, OSCE |
| Wade S.W.T. et al.^24^ | 5^th^ and 6^th^ year medical students participating in eLearning radiology education teaching imaging indications, anatomy, basic interpretation, risks, and radiation protection. Comparison of interactive vs non-interactive eLearning. | An, In, Bi, Rrp | MCQ, DD |
| ***Combined junior and senior medical student studies (n=3)*** | | | |
| Velan G.M. et al.^21^ | 1^st^, 3^rd^-6^th^ year medical students receiving teaching on imaging indications. Comparison of teaching via interactive eLearning modules vs written information (PDF files). | In | MCQ |
| Vollman A. et al.^86^ | Teaching 2^nd^ and 3^rd^ year students’ anatomy using US and or MRI. Comparison of teaching with fused ultrasound and MRI images versus ultrasound images only. | An | MCQ |
| Willis M.H. et al.^26^ | Teaching 3^rd^ and 4^th^ year students imaging indications using eLearning ‘TEACHES’ program. Comparison using interactive eLearning modules versus no access to modules on radiology clerkships. | In | MCQ |
| Wong V. et al.^23^ | Teaching 1^st^-4^th^ year students using eLearning to teach imaging indications, anatomy, risks, and basic interpretation. Comparison of interactive versus non interactive eLearning. | An, In, Bi, Rrp | MCQ, DD, RA |
| ***Student Cohort Experience Not Specified (n=3)*** | | | |
| Ebert J. and Tutschek B.^25^ | Teaching students foetal brain anatomy and brain malformations on foetal ultrasound. Comparison of using virtual reality versus a 1-hour video lecture. | An, Bi | MCQ |
| Petersson H. et al.^40^ | Teaching anatomy using cross sectional imaging. Comparison of using eLearning (Educational Virtual Anatomy / EVA program) versus no access to eLearning. | An | N/S |
| Yuan Q. et al.^87^ | Teaching students’ anatomy. Comparison of eLearning (fusing cross sectional imaging and anatomy cross sections) versus lectures. | An | SA, MOD |

***An*** *– Anatomy*

***In*** *– Indications*

***Bi*** *– Basic interpretation / radiological pathology.*

***Rrp*** *– Risks and radiation protection.*

***Us*** *– ultrasound scanning*

***DD*** *– drag and drop (including identifying regions of interest on images).*

***MCQ*** *– multiple choice*

***MOD*** *– using imaging to create a virtual model for future practice.*

***N/S*** *– not specified.*

***OR*** *– oral*

***OSCE*** *– objective structure clinical examination*

***RA*** *– ranking or ordering responses.*

***SA*** *– short answer*

***US*** *– ultrasound scanning*
